# Crystal structures and Hirshfeld surface analyses of bis­(4,5-di­hydro­furan-2-yl)di­methyl­silane and (4,5-di­hydro­furan-2-yl)(meth­yl)di­phenyl­silane

**DOI:** 10.1107/S2056989021012548

**Published:** 2022-01-01

**Authors:** Annika Schmidt, Anna Krupp, Eva Rebecca Barth, Carsten Strohmann

**Affiliations:** a Technische Universität Dortmund, Fakultät für Chemie und chemische Biologie, Anorganische Chemie, Otto-Hahn-Strasse 6, 44227 Dortmund, Germany

**Keywords:** crystal structure, di­hydro­furanyl group (DHF), Hirshfeld surface analysis, di­hydro­furylsilanes

## Abstract

The title compounds, C_10_H_16_O_2_Si (**1**) and C_17_H_18_OSi (**2**), are classified as di­hydro­furylsilanes, which show great potential as building blocks for various functionalized silanes. They both crystallize in the space group *P*  in the triclinic crystal system. Analyses of the Hirshfeld surfaces show packing-determining inter­actions for both compounds, resulting in a polymeric chain along the [011] for silane **1** and a layered-inter­connected structure along the *b*-axis direction for silane **2**.

## Abstract 2

The mol­ecular and crystal structures of two di­hydro­furylsilanes are reported. A Hirshfeld surface analysis was performed to investigate the inter­molecular inter­actions.

## Chemical context

Di­hydro­furylsilanes are inter­esting starting materials for tailor-made silicon compounds. First presented in the 1980s by Lukevics (Lukevics *et al.*, 1985 ▸), they turned out to be versatile building blocks for multiple silicon compound classes. Tetra­substituted silicon compounds are obtainable as a result of the excellent nature of the di­hydro­furyl (DHF) substituent as a leaving group in various nucleophilic substitutions at the silicon atom. Si—C(DHF) bond cleavages under substitution of the di­hydro­furyl group was observed for the reactions with C-nucleophiles (*e.g.* organolithium compounds) (Gevorgyan *et al.*, 1992 ▸), H-nucleophiles (*e.g.* LiAlH_4_, NaH, NaBH_4_) (Gevorgyan *et al.*, 1989 ▸, 1990 ▸), O-nucleophiles (*e.g. t*-butanol) and N-nucleophiles [*e.g.* LiN(Et)_2_] (Lukevics *et al.*, 1997 ▸). By means of this efficient pathway, a noteworthy approach to penta­coordinated organyl silatranes has been made (Gevorgyan *et al.*, 1997 ▸), as well as for (α-amino­meth­yl)silanes (Labrecque *et al.*, 1994 ▸). Along with their easy preparation and hydrolytical and chromatographical stability (Gevorgyan *et al.*, 1997 ▸), di­hydro­furylsilanes offer the potential to be useful reagents as protecting groups for the synthesis of amino­methyl­silaza­nes (Colquhoun *et al.*, 2011 ▸; Colquhoun & Strohmann, 2012 ▸). 
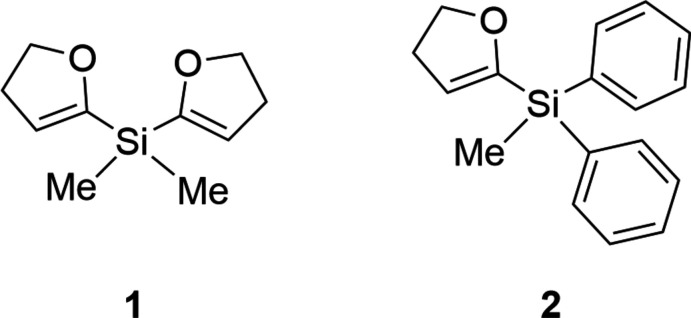


Herein, we report the structures of two further di­hydro­furylsilanes, bis­(4,5-di­hydro­furan-2-yl)di­methyl­silane (**1**) and (4,5-di­hydro­furan-2-yl)(meth­yl)di­phenyl­silane (**2**) and their structural analysis, supplemented by a Hirshfeld surface analysis.

## Structural commentary

**Figure 1:**
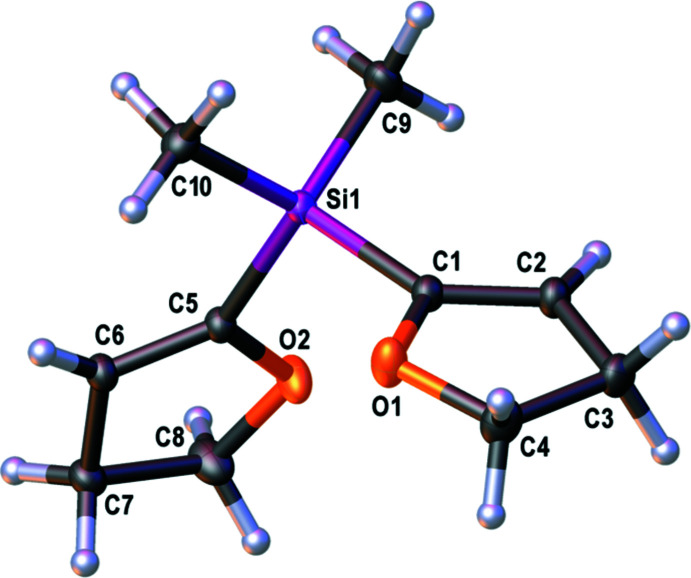
The mol­ecular structure of compound **1** with displacement ellipsoids drawn at the 50% probability level.

The mol­ecular structure of **1** is given in Fig. 1 ▸ and selected bond lengths and angles are given in Table 1 ▸. Compound **1** shows *C*_2_ mol­ecular symmetry. The lengths of the Si—C(DHF) bonds are similar but slightly longer than the lengths of the Si—C(Me) bonds. However, all bonds have characteristic dimensions (Allen *et al.*, 1987 ▸). Furthermore, the length of the C=C double bond corresponds well with literature values (Allen *et al.*, 1987 ▸) and is clearly shortened in comparison to the C—C single bonds in the di­hydro­furanyl substituent. The silicon atom is tetra­hedrally surrounded by its substituents, however slightly distorted as evident from the slight deviations from the ideal angle of 109.47°. These deviations are congruent with a former publication on di­hydro­furylsilanes (Krupp *et al.*, 2020 ▸). Both DHF planes display planarity while the C1–C4/O1 ring has an r.m.s. deviation of 0.0197 Å from an ideal least-squares plane with atom C4 showing the largest deviation of −0.0269 (2) Å. The C5–C8/O2 ring deviates more strongly from an ideal least-square plane with an r.m.s. deviation of 0.0608 Å, with the C8 atom deviating the most by 0.0838 (2) Å. The angle between the normals of the least-squares planes through the DHF rings is 78.943 (15)°.

**Figure 2:**
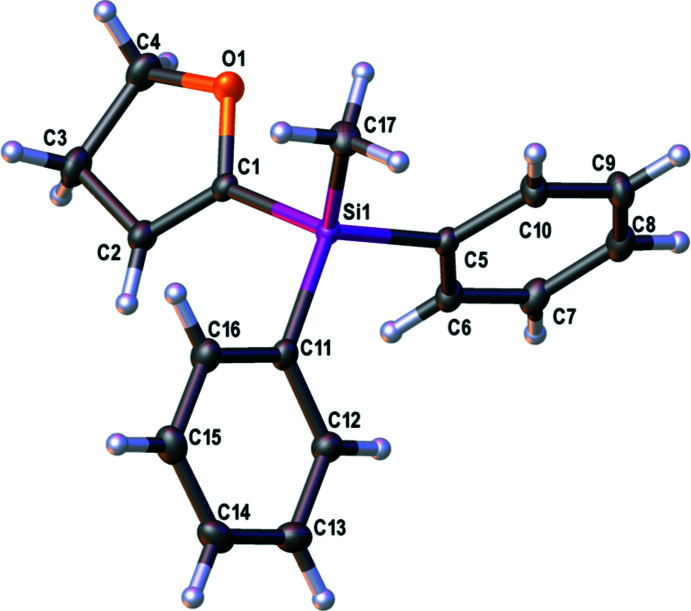
The mol­ecular structure of compound **2** with displacement ellipsoids drawn at the 50% probability level.

The mol­ecular structure of **2** is given in Fig. 2 ▸ and selected bond lengths and angles are given in Table 2 ▸. The length of the Si—C(DHF) bond is in the range of the lengths of the Si—C(Ph) bonds, which are again slightly longer than the Si—C(Me) bond. The bond lengths of the di­hydro­furan ring are consistent with those of structure **1**. Again, a slightly distorted tetra­hedral environment at the silicon atom is observed. The DHF ring is less planar than the phenyl rings, with an r.m.s. deviation from the least-squares plane of 0.0426 Å with the C4 atom having the largest deviation of −0.0582 (7) Å. The phenyl rings show r.m.s. deviations of 0.0066 and 0.0047 Å. The angle between the normals of the least-squares planes of the DHF ring and the C5–C10 phenyl ring is 87.68 (4)° and the angle between the normals of the least-squares planes of the phenyl rings is 60.03 (4)°.

## Supra­molecular features

**Figure 3:**
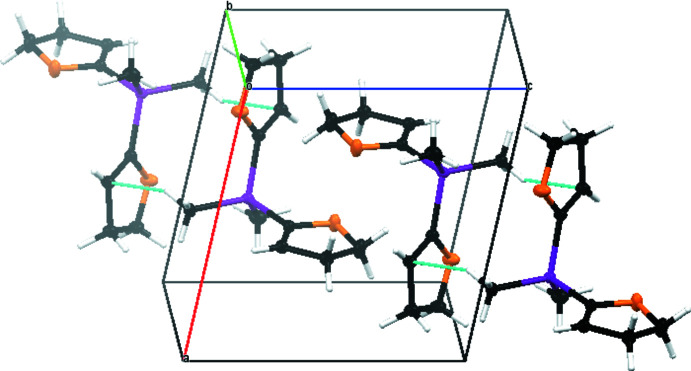
The crystal packing of compound **1**. C—H⋯C van der Waals inter­actions are shown as dashed lines.

**Figure 4:**
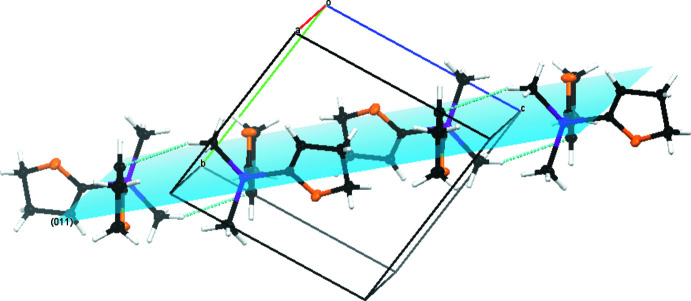
The crystal packing of compound **1** with the (011) plane shaded in blue. C—H⋯C van der Waals inter­actions are shown as dashed lines.

**Figure 5:**
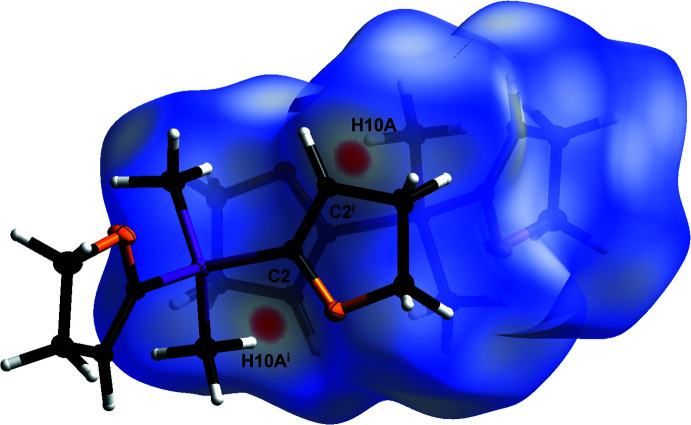
Hirshfeld surface analysis of **1** showing close contacts in the crystal. The van der Waals inter­action between carbon atom C2 and the H10*A* hydrogen atom is labeled. [Symmetry code: (i) −*x* + 1, −*y* + 2, −*z*].

**Figure 6:**
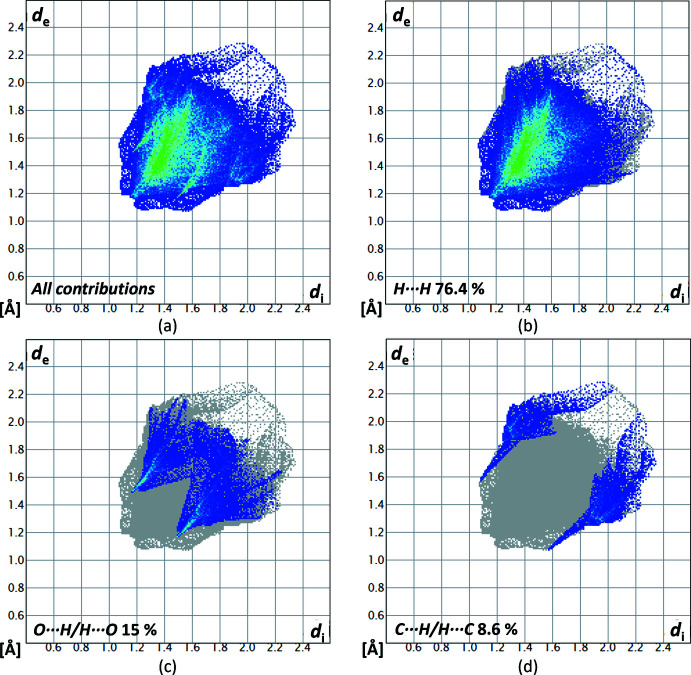
Two-dimensional fingerprint plots for compound **1**, showing (*a*) all contributions, and (*b*)–(*d*) delineated showing the contributions of atoms within specific inter­acting pairs (blue areas).

The crystal packing of compound **1** is defined by C10—H10*A*⋯C2^i^ van der Waals inter­actions as can be seen in Fig. 3 ▸. The inter­actions show relatively large distances [C10⋯C2^i^ = 3.6208 (5) Å, H10*A*⋯C2^i^ = 2.752 (10) Å and C10—H10*A*⋯C2^i^ = 148.4 (8)°; symmetry code: (i) −*x* + 1, −*y* + 2, −*z*]. As a result of the inter­actions between carbon atom C2 and hydrogen atom H10*A*, a polymeric chain structure along the [011] direction is formed (Fig. 4 ▸). The inter­actions can be displayed by a Hirshfeld surface analysis (Spackman & Jayatilaka, 2009 ▸) generated by *CrystalExplorer21* (Spackman *et al.*, 2021 ▸), here indicated by the red spots (Fig. 5 ▸). The Hirshfeld surface mapped over *d*_norm_ is in the range from −0.0783 to 1.0981 a.u. The contributions of the different types of inter­molecular inter­actions for **1** are shown in the two-dimensional fingerprint plots (McKinnon *et al.*, 2007 ▸) in Fig. 6 ▸. The contribution of the H⋯ H inter­actions, with a value of 76.4%, has the largest share of the crystal packing of **1**. The O⋯H/H⋯O inter­actions have a smaller share with a 15% contribution and the C⋯H/H⋯C inter­actions with a 8.6% contribution. Both heteronuclear inter­actions appear as spikes.

**Figure 7:**
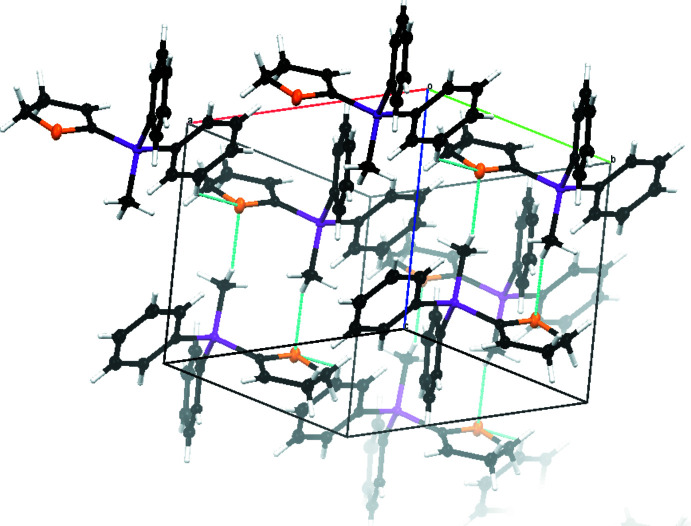
The crystal packing of compound **2**. C—H⋯O hydrogen bonds are shown as dashed lines.

**Figure 8:**
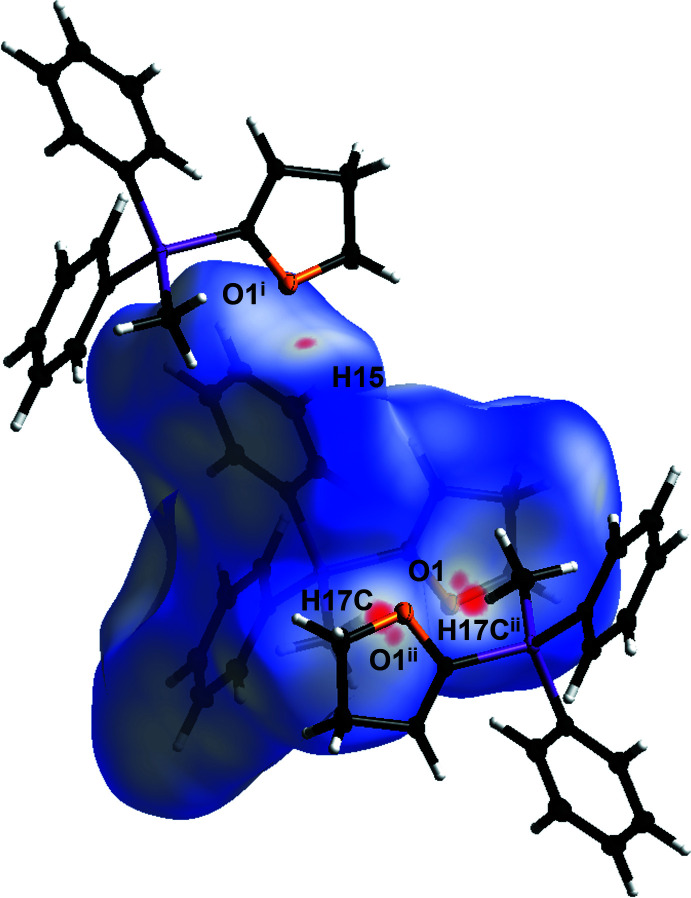
Hirshfeld surface analysis of **2** showing close contacts in the crystal. The weak hydrogen bond between oxygen atom O1 and the H15 hydrogen atom, as well as the weak hydrogen bonds between the oxygen atom O1 and the H17*C* hydrogen atom are labeled. [Symmetry codes: (i) *x*, *y* + 1, *z*; (ii) −*x* + 2, −*y* + 1, −*z* + 1].

**Figure 9:**
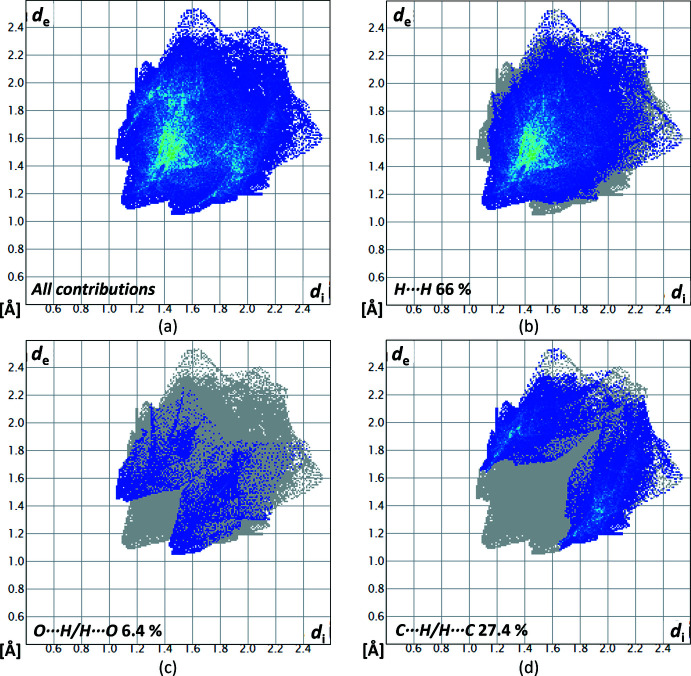
Two-dimensional fingerprint plots for compound **2**, showing (*a*) all contributions, and (*b*)–(*d*) delineated showing the contributions of atoms within specific inter­acting pairs (blue areas).

The structure of compound **2** is more strongly defined by C—H⋯O hydrogen bonds (Fig. 7 ▸, Table 3 ▸). Two different layers are formed along the *b*-axis direction and inter­connected by hydrogen bonds between the O1 atom and the H17*C* atom. An additional inter­action in each of the layers is observed by C—H⋯O hydrogen bonds between the O1 atom and the H15 atom. The C17—H17*C*⋯O1^ii^ hydrogen bond can be described by the 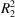 (10) graph-set motif and the C15—H15⋯O1^i^ hydrogen bond by the 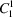 (7) graph-set motif (Etter *et al.*, 1990 ▸). Both C—H⋯O hydrogen bonds can be identified as weak inter­actions according to Desiraju & Steiner (2001 ▸). A Hirshfeld analysis, carried out analogously as for structure **1**, was used to further study the crystal packing. In Fig. 8 ▸, the nearest contacts are shown in red. The Hirshfeld surface mapped over *d*_norm_ is in the range from −0.0717 to 1.0768 a.u. By analysis of the two-dimensional fingerprint plots (Fig. 9 ▸), again, the biggest contribution to the crystal packing can be assigned to H⋯H inter­actions (66%). Although the closest contacts were identified as C—H⋯O hydrogen bonds, O⋯H/H⋯O inter­actions contribute only 6.4% to the crystal packing, while C⋯H/H⋯C inter­actions have a larger share of 27%.

## Database survey

A search of the Cambridge Crystallographic Database (WebCSD, November 2021; Groom *et al.*, 2016 ▸) for 2-(4,5-di­hydro­fur­yl)silanes revealed solely the structures of tris­(4,5-di­hydro­furan-2-yl)methyl­silane and tris­(4,5-di­hydro­furan-2-yl)phenyl­silane published by our group previously (Krupp *et al.*, 2020 ▸). A more extended search for 3-(4,5-di­hydro­fur­yl)silane gave some structures with substituted di­hydro­furan rings, such as [4-(4-fluoro­phen­yl)-5-(4-nitro­phen­yl)-4,5-di­hydro­furan-3-yl](trimeth­yl)silane (JIVLIM; Li & Zhang, 2018 ▸), *rac*-5-phenyl-4-(*t*-butyl­diphenyl­sil­yl)-2,3-di­hydro­furan-2-carb­oxy­lic acid ethyl ester (PUXCAM; Evans *et al.*, 2001 ▸) and (1′*S*,2*R*)-5-methyl-4-(*t*-butyl­diphenyl­sil­yl)-2,3-di­hydro­furan-2-carb­oxy­lic acid (1′-phenyl­eth­yl)amide (PUXCEQ; Evans *et al.*, 2001 ▸). Contrary to the here and previously presented 2-(4,5-di­hydro­fur­yl)silanes (Krupp *et al.*, 2020 ▸), the published 3-(4,5-di­hydro­fur­yl)silanes do not show an elongated Si—C(DHF) bond in comparison to the other substituents at the silicon atom. This can be attributed to the changed connection on the DHF ring. The slightly distorted tetra­hedral silicon atom can be observed in all structures as well as the shortened C=C double bond in the DHF ring.

## Synthesis and crystallization

Bis(4,5-di­hydro­furan-2-yl)di­methyl­silane (**1**) as already described by Lukevics and co-workers (Lukevics *et al.*, 1985 ▸) was synthesized by adding ^*t*^BuLi (1.9 *M* in pentane, 8.16 mL, 15.5 mmol, 2.0 eq.) to a solution of 2,3-di­hydro­furan (1.09 g, 15.5 mmol, 2.0 eq.) in diethyl ether at 243 K and subsequent stirring for an hour. Di­chloro­dimethyl­silane (1.00 g, 7.75 mmol, 1.0 eq.) was added at 243 K and warmed to room temperature under stirring for 2 h. All solids were filtered off inertly and all volatile components were removed *in vacuo*. After cleaning by Kugelrohr distillation (temperature: 373 K, pressure: 2.1 × 10^−1^ mbar), bis­(4,5-di­hydro­furan-2-yl)di­methyl­silane (**1**) (1.50 g, 7.65 mmol, 99%) was obtained as a colorless oil. By crystallization from diethyl ether at 193 K, colorless blocks were obtained.

^1^H NMR (400.25 MHz, C_6_D_6_): *δ* = 0.39 [*s*, 6H; Si(C*H*_3_)_2_], 2.27 [*dt*, ^3^ *J*_HH_ = 2.57 Hz, ^3^ *J*_HH_ = 9.78 Hz, 4H; Si(CCHC*H*_2_)_2_], 4.07 [*t*, ^3^ *J*_HH_ = 9.78 Hz, 4H; Si(COC*H*_2_)_2_], 5.31 [*t*, ^3^ *J*_HH_ = 2.57 Hz, 2H; Si(CC*H*)_2_] ppm.

{^1^H}^13^C NMR (100.6 MHz, C_6_D_6_): *δ* = −3.8 [2C; (Si*C*H_3_)_2_], 31.6 [2C; Si(CCH*C*H_2_)_2_)], 71.8 [2C; Si(CO*C*H_2_)_2_], 113.4 [2C; Si(CO*C*H_2_)_2_], 160.0 [2C; Si(*C*O)_2_] ppm.

{^1^H}^29^Si NMR (79.52 MHz, C_6_D_6_): *δ* = −22.29 [1Si; *Si*(DHF)_2_] ppm.

GC/EI–MS: *t*_R_ = 3.94 min [353 K (1 min) – 40 K min^−1^ – 543 K (5.5 min)]; *m*/*z* (%): 196 (94) [*M* ^+^], 181 (2) [(*M* − Me)^+^], 167 (14) [(*M* − CHO)^+^], 153 (40) [(*M* − C_2_H_3_O)^+^], 97 (100) [(SiDHF)^+^].

(4,5-Di­hydro­furan-2-yl)(meth­yl)di­phenyl­silane (**2**), already described by Tsai and co-workers (Tsai *et al.*, 1992 ▸) by cycliz­ation of a halo­acyl­silane, was synthesized analogously to **1**. ^*t*^BuLi (1.90 *M* in pentane, 21.5 mmol, 11.3 mL, 1.00 eq.) was slowly added dropwise to a solution of 2,3-di­hydro­furan (1.51 g, 21.5 mmol, 1.00 eq.) in diethyl ether (80 mL) at 243 K and the reaction solution was stirred for 1h at this temperature. Methyl­diphenyl­chloro­silane (5.00 g, 21.5 mmol, 1.00 eq.) was then added at 243 K and the reaction solution was stirred at room temperature overnight. All solids were separated by inert filtration and the solvent was removed *in vacuo*. The residue was purified by Kugelrohr distillation (temperature: 453 K, pressure: 2.0 × 10^−1^ mbar) and the product **2** (5.11 g, 19.2 mmol, 89%) was obtained as a colorless liquid. By crystallization from pentane at 193 K, colorless platelets were obtained.

^1^H NMR (400.25 MHz, C_6_D_6_): δ = 0.69 (*s*, 3H; SiC*H*_3_), 2.24 (*dt*, ^3^ *J*_HH_ = 2.57Hz, ^3^ *J*_HH_ = 9.66 Hz, 2H; SiCCHC*H*_2_), 4.07 (*t*, ^3^ *J*_HH_ = 9.66 Hz, 2H; SiCOC*H*_2_), 5.20 (*t*, ^3^ *J*_HH_ = 2.57 Hz, 1H; SiCC*H*), 7.18–7.21 (*m*, 6H; C*H*_*ortho*,*para*_), 7.71–7.73 (*m*, 4H; C*H_meta_*) ppm.

{^1^H}^13^C NMR (100.65 MHz, C_6_D_6_): δ = −3.9 (1C; Si*C*H_3_), 31.3 (1C; SiCCH*C*H_2_), 71.1 (1C; SiCO*C*H_2_), 115.5 (1C; SiC*C*H), 128.5 (2C, *C_ortho_*), 130.2 (1C, *C_para_*), 135.8 (2C, *C_meta_*), 135.8 (1C, *C_ipso_*), 160.2 (1C, Si*C*O) ppm.

{^1^H}^29^Si NMR (79.52 MHz, C_6_D_6_): δ = −19.51 (*s*, 1Si; *Si*DHF) ppm.

GC/EI–MS: *t*_R_ = 5.97 min [353 K (1 min) – 40 K min^−1^ – 543 K (5.5 min)]; *m*/*z* (%): 266 (100) [*M* ^+^], 251 (33) [(*M* − Me)^+^], 238 (27) [(*M* − C_2_H_4_)^+^], 222 (20) [(*M* − C_2_H_4_O)^+^], 197 (75) [(*M* − DHF)^+^], 105 (52) [(SiPh)^+^], 77 (6) [(Ph)^+^].

## Refinement

Crystal data, data collection and structure refinement details are summarized in Table 4 ▸. H atoms were positioned geometrically (C—H = 0.95–1.00 Å) and were refined using a riding model, with *U*_iso_(H) = 1.2*U*_eq_(C) for CH_2_ and CH hydrogen atoms and *U*_iso_(H) = 1.5*U*_eq_(C) for CH_3_ hydrogen atoms. Hydrogen atoms H2 and H6 for compound **1** and H2, H15 and H17*C* for compound **2** were refined independently.

## Supplementary Material

Crystal structure: contains datablock(s) 1, 2. DOI: 10.1107/S2056989021012548/vm2257sup1.cif

Structure factors: contains datablock(s) 1. DOI: 10.1107/S2056989021012548/vm22571sup2.hkl

Structure factors: contains datablock(s) 2. DOI: 10.1107/S2056989021012548/vm22572sup2.hkl

CCDC references: 2124286, 2124285

Additional supporting information:  crystallographic information; 3D view; checkCIF report

## Figures and Tables

**Table 1:** Selected geometric parameters for compound **1** (Å, °)

Si1—C1	1.8742 (3)	C1—Si1—C5	108.189 (12)
Si1—C5	1.8693 (3)	C1—Si1—C9	106.801 (14)
Si1—C9	1.8631 (3)	C1—Si1—C10	109.191 (13)
Si1—C10	1.8579 (3)	C5—Si1—C9	110.770 (15)
		C5—Si1—C10	108.128 (13)
C1—C2	1.3370 (4)	C9—Si1—C10	113.628 (16)
C3—C4	1.5331 (5)		
C5—C6	1.3409 (4)		
C7—C8	1.5298 (5)		

**Table 2:** Selected geometric parameters for compound **2** (Å, °)

Si1—C1	1.8742 (10)	C1—Si1—C5	108.44 (4)
Si1—C5	1.8721 (9)	C1—Si1—C11	105.51 (4)
Si1—C11	1.8713 (10)	C1—Si1—C17	109.26 (5)
Si1—C17	1.8591 (11)	C5—Si1—C11	113.08 (4)
		C5—Si1—C17	110.49 (5)
C1—C2	1.3356 (14)	C11—Si1—C17	109.88 (59
C3—C4	1.5416 (17)		

**Table 3:** Hydrogen-bond geometry (Å, °) for **2** [Chem scheme1]

*D*—H⋯*A*	*D*—H	H⋯*A*	*D*⋯*A*	*D*—H⋯*A*
C15—H15⋯O1^i^	0.969 (16)	2.640 (16)	3.3422 (13)	129.6 (12)
C17—H17*C*⋯O1^ii^	0.992 (19)	2.584 (19)	3.5168 (14)	156.5 (15)

Symmetry codes: (i) x, y+1, z; (ii) -x+2, -y+1, -z+1.

**Table 4:** Experimental details

	**1**	**2**
Crystal data		
Chemical formula	C_10_H_16_O_2_Si	C_17_H_18_OSi
*M*_r_	196.32	266.40
Crystal system, space group	Triclinic, *P*\overline{1}	Triclinic, *P*\overline{1}
Temperature (K)	100	100
*a*, *b*, *c* (Å)	8.2422 (3), 8.3075 (4), 8.2940 (4)	8.7737 (4), 9.1715 (4), 9.8130 (4)
α, β, γ (°)	94.149 (2), 103.012 (1), 104.909 (1)	102.219 (2), 90.613 (2), 110.280 (2)
*V* (Å^3^)	529.55 (4)	720.85 (6)
*Z*	2	2
Radiation type	Mo *K*α	Mo *K*α
μ (mm^−1^)	0.19	0.15
Crystal size (mm)	0.72 × 0.66 × 0.59	0.51 × 0.19 × 0.07

Data collection		
Diffractometer	Bruker D8 Venture	Bruker D8 Venture
Absorption correction	Multi-scan (*SADABS*; Krause *et al.*, 2015 ▸)	Multi-scan (*SADABS*; Krause *et al.*, 2015 ▸)
*T*_min_, *T*_max_	0.519, 0.576	0.713, 0.747
No. of measured, independent and observed [*I* > 2σ(*I*)] reflections	256332, 11412, 10306	20409, 5826, 4937
*R*_int_	0.032	0.030
(sin θ/λ)_max_ (Å^−1^)	1.089	0.787

Refinement		
*R*[*F* ^2^ > 2σ(*F* ^2^)], *wR*(*F* ^2^), *S*	0.026, 0.089, 1.06	0.040, 0.105, 1.06
No. of reflections	11412	5826
No. of parameters	182	244
H-atom treatment	H atoms treated by a mixture of independent and constrained refinement.	H atoms treated by a mixture of independent and constrained refinement.
Δρ_max_, Δρ_min_ (e Å^−3^)	0.68, −0.32	0.48, −0.27

Computer programs: *APEX2* (Bruker, 2018 ▸), *SAINT* (Bruker, 2016 ▸), *SHELXS* (Sheldrick, 2008 ▸), *SHELXL2014/7* (Sheldrick, 2015 ▸), *OLEX2* (Dolomanov *et al.*, 2009 ▸), *publCIF* (Westrip, 2010 ▸) and *Mercury* (Macrae *et al.*, 2020 ▸).

## supplementary crystallographic information

### Bis(4,5-dihydrofuran-2-yl)dimethylsilane (1) Crystal data

**Table:**

C_10_H_16_O_2_Si	*Z* = 2
*M**_r_* = 196.32	*F*(000) = 212
Triclinic, *P*1	*D*_x_ = 1.231 Mg m^−^^3^
*a* = 8.2422 (3) Å	Mo *K*α radiation, λ = 0.71073 Å
*b* = 8.3075 (4) Å	Cell parameters from 8689 reflections
*c* = 8.2940 (4) Å	θ = 2.5–20.9°
α = 94.149 (2)°	µ = 0.19 mm^−^^1^
β = 103.012 (1)°	*T* = 100 K
γ = 104.909 (1)°	Block, colourless
*V* = 529.55 (4) Å^3^	0.72 × 0.66 × 0.59 mm

### Bis(4,5-dihydrofuran-2-yl)dimethylsilane (1) Data collection

**Table:**

Bruker D8 Venture diffractometer	11412 independent reflections
Radiation source: microfocus sealed X-ray tube, Incoatec Iµs	10306 reflections with *I* > 2σ(*I*)
Mirror optics monochromator	*R*_int_ = 0.032
Detector resolution: 7.9 pixels mm^-1^	θ_max_ = 50.7°, θ_min_ = 2.6°
ω and φ scans	*h* = −16→17
Absorption correction: multi-scan (SADABS; Krause et al., 2015)	*k* = −18→18
*T*_min_ = 0.519, *T*_max_ = 0.576	*l* = −18→18
256332 measured reflections	

### Bis(4,5-dihydrofuran-2-yl)dimethylsilane (1) Refinement

**Table:**

Refinement on *F*^2^	Primary atom site location: iterative
Least-squares matrix: full	Hydrogen site location: difference Fourier map
*R*[*F*^2^ > 2σ(*F*^2^)] = 0.026	H_atoms_treated_by_a_mixture_of_independent_and_constrained_refinement.
*wR*(*F*^2^) = 0.089	*w* = 1/[σ^2^(*F*_o_^2^) + (0.0535*P*)^2^ + 0.0207*P*] where *P* = (*F*_o_^2^ + 2*F*_c_^2^)/3
*S* = 1.06	(Δ/σ)_max_ = 0.002
11412 reflections	Δρ_max_ = 0.68 e Å^−^^3^
182 parameters	Δρ_min_ = −0.32 e Å^−^^3^
0 restraints	

### Bis(4,5-dihydrofuran-2-yl)dimethylsilane (1) Special details

**Table:**

Geometry. All esds (except the esd in the dihedral angle between two l.s. planes) are estimated using the full covariance matrix. The cell esds are taken into account individually in the estimation of esds in distances, angles and torsion angles; correlations between esds in cell parameters are only used when they are defined by crystal symmetry. An approximate (isotropic) treatment of cell esds is used for estimating esds involving l.s. planes.

### Bis(4,5-dihydrofuran-2-yl)dimethylsilane (1) Fractional atomic coordinates and isotropic or equivalent isotropic displacement parameters (Å^2^)

**Table:**

	*x*	*y*	*z*	*U*_iso_*/*U*_eq_	
Si1	0.64185 (2)	0.82439 (2)	0.20897 (2)	0.01354 (2)	
O1	0.28272 (3)	0.68623 (3)	0.09023 (4)	0.02227 (4)	
O2	0.70145 (4)	0.71875 (3)	0.52219 (3)	0.02213 (4)	
C1	0.41042 (3)	0.81964 (3)	0.19462 (3)	0.01504 (3)	
C2	0.34535 (4)	0.92843 (4)	0.26763 (4)	0.01740 (4)	
C3	0.14995 (4)	0.87390 (4)	0.21112 (4)	0.02057 (4)	
H3A	0.0906 (12)	0.8525 (12)	0.3024 (12)	0.033 (2)*	
H3B	0.1063 (12)	0.9549 (11)	0.1543 (11)	0.032 (2)*	
C4	0.11591 (4)	0.70688 (5)	0.10008 (5)	0.02242 (5)	
H4A	0.0538 (11)	0.6100 (11)	0.1453 (11)	0.032 (2)*	
H4B	0.0486 (12)	0.6963 (12)	−0.0129 (12)	0.037 (2)*	
C5	0.70144 (3)	0.67768 (3)	0.35736 (3)	0.01419 (3)	
C6	0.73403 (4)	0.52967 (3)	0.32841 (3)	0.01609 (4)	
C7	0.75301 (4)	0.44890 (4)	0.48628 (4)	0.01889 (4)	
H7A	0.8533 (11)	0.4108 (11)	0.5151 (11)	0.0284 (18)*	
H7B	0.6498 (10)	0.3515 (11)	0.4771 (10)	0.0251 (17)*	
C8	0.75865 (6)	0.59310 (5)	0.61494 (4)	0.02397 (6)	
H8A	0.6831 (12)	0.5602 (11)	0.6876 (12)	0.033 (2)*	
H8B	0.8762 (14)	0.6488 (14)	0.6853 (14)	0.048 (3)*	
C9	0.77560 (4)	1.04490 (4)	0.28985 (5)	0.02162 (5)	
H9A	0.7566 (12)	1.0847 (12)	0.3964 (12)	0.037 (2)*	
H9B	0.9008 (14)	1.0571 (13)	0.3185 (14)	0.048 (3)*	
H9C	0.7555 (14)	1.1226 (14)	0.2124 (13)	0.047 (3)*	
C10	0.66177 (4)	0.74698 (4)	0.00048 (4)	0.01893 (4)	
H10A	0.6108 (13)	0.8064 (13)	−0.0859 (13)	0.040 (2)*	
H10B	0.6006 (14)	0.6296 (14)	−0.0355 (13)	0.045 (3)*	
H10C	0.7765 (13)	0.7669 (12)	−0.0041 (12)	0.036 (2)*	
H6	0.7325 (10)	0.4798 (10)	0.2272 (10)	0.0225 (16)*	
H2	0.4137 (12)	1.0273 (11)	0.3403 (11)	0.033 (2)*	

### Bis(4,5-dihydrofuran-2-yl)dimethylsilane (1) Atomic displacement parameters (Å^2^)

**Table:**

	*U*^11^	*U*^22^	*U*^33^	*U*^12^	*U*^13^	*U*^23^
Si1	0.01424 (3)	0.01281 (3)	0.01491 (3)	0.00527 (2)	0.00450 (2)	0.00244 (2)
O1	0.01651 (7)	0.02070 (9)	0.02720 (10)	0.00574 (6)	0.00287 (7)	−0.00545 (7)
O2	0.03579 (12)	0.02135 (9)	0.01466 (7)	0.01572 (9)	0.00835 (7)	0.00268 (6)
C1	0.01497 (7)	0.01501 (7)	0.01637 (8)	0.00611 (6)	0.00417 (6)	0.00253 (6)
C2	0.01676 (8)	0.01709 (8)	0.01982 (9)	0.00723 (7)	0.00523 (7)	0.00100 (7)
C3	0.01692 (9)	0.02220 (11)	0.02551 (12)	0.00930 (8)	0.00669 (8)	0.00381 (9)
C4	0.01543 (9)	0.02642 (13)	0.02320 (11)	0.00544 (8)	0.00243 (8)	−0.00117 (9)
C5	0.01579 (7)	0.01407 (7)	0.01381 (7)	0.00590 (6)	0.00406 (6)	0.00171 (5)
C6	0.01916 (9)	0.01436 (8)	0.01617 (8)	0.00689 (6)	0.00502 (7)	0.00142 (6)
C7	0.02225 (10)	0.01595 (8)	0.02068 (10)	0.00752 (7)	0.00644 (8)	0.00561 (7)
C8	0.03680 (17)	0.02108 (11)	0.01494 (9)	0.01061 (11)	0.00498 (9)	0.00432 (8)
C9	0.02043 (10)	0.01447 (9)	0.02932 (13)	0.00399 (7)	0.00669 (9)	0.00107 (8)
C10	0.02063 (10)	0.02226 (10)	0.01611 (9)	0.00769 (8)	0.00686 (7)	0.00360 (7)

### Bis(4,5-dihydrofuran-2-yl)dimethylsilane (1) Geometric parameters (Å, º)

**Table:**

Si1—C1	1.8742 (3)	C4—H4B	0.961 (10)
Si1—C5	1.8693 (3)	C5—C6	1.3409 (4)
Si1—C9	1.8631 (3)	C6—C7	1.5093 (4)
Si1—C10	1.8579 (3)	C6—H6	0.904 (8)
O1—C1	1.3915 (4)	C7—H7A	0.947 (9)
O1—C4	1.4479 (4)	C7—H7B	0.996 (8)
O2—C5	1.3849 (3)	C7—C8	1.5298 (5)
O2—C8	1.4525 (4)	C8—H8A	0.965 (9)
C1—C2	1.3370 (4)	C8—H8B	0.984 (11)
C2—C3	1.5075 (4)	C9—H9A	0.980 (10)
C2—H2	0.945 (9)	C9—H9B	0.982 (11)
C3—H3A	0.992 (9)	C9—H9C	0.961 (11)
C3—H3B	0.948 (9)	C10—H10A	0.978 (10)
C3—C4	1.5331 (5)	C10—H10B	0.965 (11)
C4—H4A	0.984 (9)	C10—H10C	0.927 (10)
			
C5—Si1—C1	108.189 (12)	C6—C5—O2	113.03 (2)
C9—Si1—C1	106.801 (14)	C5—C6—C7	109.72 (2)
C9—Si1—C5	110.770 (15)	C5—C6—H6	125.3 (5)
C10—Si1—C1	109.191 (13)	C7—C6—H6	124.7 (5)
C10—Si1—C5	108.128 (13)	C6—C7—H7A	114.8 (5)
C10—Si1—C9	113.628 (16)	C6—C7—H7B	110.3 (5)
C1—O1—C4	107.60 (2)	C6—C7—C8	101.18 (2)
C5—O2—C8	107.15 (2)	H7A—C7—H7B	107.9 (7)
O1—C1—Si1	117.093 (19)	C8—C7—H7A	111.6 (5)
C2—C1—Si1	129.94 (2)	C8—C7—H7B	110.9 (5)
C2—C1—O1	112.95 (2)	O2—C8—C7	106.95 (2)
C1—C2—C3	110.20 (3)	O2—C8—H8A	107.7 (6)
C1—C2—H2	124.1 (5)	O2—C8—H8B	106.9 (6)
C3—C2—H2	125.7 (5)	C7—C8—H8A	113.7 (6)
C2—C3—H3A	114.7 (5)	C7—C8—H8B	113.2 (6)
C2—C3—H3B	111.8 (5)	H8A—C8—H8B	108.0 (8)
C2—C3—C4	101.57 (2)	Si1—C9—H9A	111.7 (5)
H3A—C3—H3B	106.4 (7)	Si1—C9—H9B	113.1 (6)
C4—C3—H3A	108.8 (5)	Si1—C9—H9C	113.1 (6)
C4—C3—H3B	113.8 (5)	H9A—C9—H9B	102.7 (8)
O1—C4—C3	107.47 (3)	H9A—C9—H9C	108.8 (8)
O1—C4—H4A	108.9 (5)	H9B—C9—H9C	106.8 (9)
O1—C4—H4B	106.6 (6)	Si1—C10—H10A	111.1 (6)
C3—C4—H4A	112.4 (5)	Si1—C10—H10B	112.9 (6)
C3—C4—H4B	116.7 (6)	Si1—C10—H10C	112.3 (6)
H4A—C4—H4B	104.5 (8)	H10A—C10—H10B	105.1 (8)
O2—C5—Si1	116.686 (18)	H10A—C10—H10C	104.8 (8)
C6—C5—Si1	130.12 (2)	H10B—C10—H10C	110.1 (8)
			
Si1—C1—C2—C3	177.42 (2)	C5—O2—C8—C7	−13.01 (4)
Si1—C5—C6—C7	−172.51 (2)	C5—C6—C7—C8	−10.06 (3)
O1—C1—C2—C3	−1.01 (4)	C6—C7—C8—O2	13.68 (4)
O2—C5—C6—C7	2.56 (4)	C8—O2—C5—Si1	−177.44 (2)
C1—Si1—C5—O2	−64.48 (2)	C8—O2—C5—C6	6.78 (4)
C1—Si1—C5—C6	110.44 (3)	C9—Si1—C1—O1	160.08 (2)
C1—O1—C4—C3	4.15 (4)	C9—Si1—C1—C2	−18.30 (3)
C1—C2—C3—C4	3.38 (4)	C9—Si1—C5—O2	52.26 (3)
C2—C3—C4—O1	−4.46 (4)	C9—Si1—C5—C6	−132.81 (3)
C4—O1—C1—Si1	179.30 (2)	C10—Si1—C1—O1	36.82 (3)
C4—O1—C1—C2	−2.04 (4)	C10—Si1—C1—C2	−141.56 (3)
C5—Si1—C1—O1	−80.64 (2)	C10—Si1—C5—O2	177.38 (2)
C5—Si1—C1—C2	100.98 (3)	C10—Si1—C5—C6	−7.70 (3)

### (4,5-Dihydrofuran-2-yl)(methyl)diphenylsilane (2) Crystal data

**Table:**

C_17_H_18_OSi	*Z* = 2
*M**_r_* = 266.40	*F*(000) = 284
Triclinic, *P*1	*D*_x_ = 1.227 Mg m^−^^3^
*a* = 8.7737 (4) Å	Mo *K*α radiation, λ = 0.71073 Å
*b* = 9.1715 (4) Å	Cell parameters from 8775 reflections
*c* = 9.8130 (4) Å	θ = 2.5–36.3°
α = 102.219 (2)°	µ = 0.15 mm^−^^1^
β = 90.613 (2)°	*T* = 100 K
γ = 110.280 (2)°	Plate, colourless
*V* = 720.85 (6) Å^3^	0.51 × 0.19 × 0.07 mm

### (4,5-Dihydrofuran-2-yl)(methyl)diphenylsilane (2) Data collection

**Table:**

Bruker D8 Venture diffractometer	5826 independent reflections
Radiation source: microfocus sealed X-ray tube, INCOATEC microfocus sealed tube, Iys 3.0	4937 reflections with *I* > 2σ(*I*)
Multilayer optics monochromator	*R*_int_ = 0.030
Detector resolution: 10.4167 pixels mm^-1^	θ_max_ = 34.0°, θ_min_ = 2.8°
φ and ω scans	*h* = −11→13
Absorption correction: multi-scan (SADABS; Krause et al., 2015)	*k* = −14→14
*T*_min_ = 0.713, *T*_max_ = 0.747	*l* = −15→15
20409 measured reflections	

### (4,5-Dihydrofuran-2-yl)(methyl)diphenylsilane (2) Refinement

**Table:**

Refinement on *F*^2^	Primary atom site location: iterative
Least-squares matrix: full	Hydrogen site location: difference Fourier map
*R*[*F*^2^ > 2σ(*F*^2^)] = 0.040	H_atoms_treated_by_a_mixture_of_independent_and_constrained_refinement.
*wR*(*F*^2^) = 0.105	*w* = 1/[σ^2^(*F*_o_^2^) + (0.0391*P*)^2^ + 0.2951*P*] where *P* = (*F*_o_^2^ + 2*F*_c_^2^)/3
*S* = 1.06	(Δ/σ)_max_ < 0.001
5826 reflections	Δρ_max_ = 0.48 e Å^−^^3^
244 parameters	Δρ_min_ = −0.27 e Å^−^^3^
0 restraints	

### (4,5-Dihydrofuran-2-yl)(methyl)diphenylsilane (2) Special details

**Table:**

Geometry. All esds (except the esd in the dihedral angle between two l.s. planes) are estimated using the full covariance matrix. The cell esds are taken into account individually in the estimation of esds in distances, angles and torsion angles; correlations between esds in cell parameters are only used when they are defined by crystal symmetry. An approximate (isotropic) treatment of cell esds is used for estimating esds involving l.s. planes.

### (4,5-Dihydrofuran-2-yl)(methyl)diphenylsilane (2) Fractional atomic coordinates and isotropic or equivalent isotropic displacement parameters (Å^2^)

**Table:**

	*x*	*y*	*z*	*U*_iso_*/*U*_eq_	
Si1	0.69854 (3)	0.37535 (3)	0.34297 (3)	0.01325 (7)	
O1	0.92417 (10)	0.22237 (9)	0.28540 (9)	0.02194 (16)	
C1	0.86366 (11)	0.32897 (11)	0.24356 (10)	0.01447 (16)	
C2	0.93945 (12)	0.38813 (13)	0.13924 (11)	0.01886 (18)	
C3	1.07424 (14)	0.32450 (15)	0.10108 (13)	0.0250 (2)	
H3A	1.182 (2)	0.410 (2)	0.1241 (18)	0.035 (4)*	
H3B	1.068 (2)	0.282 (2)	0.0052 (19)	0.037 (4)*	
C4	1.04574 (14)	0.20014 (14)	0.19082 (13)	0.0241 (2)	
H4A	1.003 (2)	0.091 (2)	0.1329 (18)	0.037 (4)*	
H4B	1.143 (2)	0.213 (2)	0.2498 (17)	0.034 (4)*	
C5	0.50711 (11)	0.19477 (11)	0.29840 (10)	0.01461 (16)	
C6	0.44747 (12)	0.11776 (12)	0.15886 (10)	0.01701 (17)	
H6	0.4976 (18)	0.1611 (17)	0.0857 (15)	0.021 (3)*	
C7	0.31274 (12)	−0.02389 (13)	0.12562 (11)	0.01983 (19)	
H7	0.276 (2)	−0.0716 (19)	0.0290 (17)	0.030 (4)*	
C8	0.23486 (13)	−0.09184 (13)	0.23187 (13)	0.0221 (2)	
H8	0.1407 (19)	−0.1954 (19)	0.2088 (16)	0.028 (4)*	
C9	0.28960 (13)	−0.01617 (13)	0.37054 (12)	0.0235 (2)	
H9	0.232 (2)	−0.063 (2)	0.4496 (17)	0.034 (4)*	
C10	0.42448 (12)	0.12571 (13)	0.40346 (11)	0.01958 (18)	
H10	0.4595 (19)	0.1741 (18)	0.5007 (16)	0.025 (4)*	
C11	0.68003 (11)	0.55198 (11)	0.28740 (10)	0.01557 (16)	
C12	0.54668 (13)	0.54371 (13)	0.20270 (11)	0.01901 (18)	
H12	0.4588 (18)	0.4466 (18)	0.1690 (15)	0.021 (3)*	
C13	0.53731 (15)	0.67991 (14)	0.16669 (12)	0.0236 (2)	
H13	0.447 (2)	0.6702 (19)	0.1106 (17)	0.029 (4)*	
C14	0.66219 (15)	0.82626 (14)	0.21337 (12)	0.0239 (2)	
H14	0.6560 (19)	0.9203 (19)	0.1908 (17)	0.029 (4)*	
C15	0.79733 (14)	0.83698 (13)	0.29579 (13)	0.0234 (2)	
C16	0.80543 (13)	0.70145 (12)	0.33290 (12)	0.02067 (19)	
H16	0.902 (2)	0.7112 (19)	0.3922 (17)	0.029 (4)*	
C17	0.76169 (14)	0.42855 (14)	0.53383 (11)	0.02152 (19)	
H17A	0.779 (2)	0.341 (2)	0.5607 (19)	0.042 (5)*	
H17B	0.678 (2)	0.459 (2)	0.5845 (19)	0.041 (5)*	
H15	0.886 (2)	0.9381 (19)	0.3294 (16)	0.028 (4)*	
H17C	0.863 (2)	0.524 (2)	0.5574 (19)	0.045 (5)*	
H2	0.9161 (19)	0.4673 (19)	0.1014 (16)	0.028 (4)*	

### (4,5-Dihydrofuran-2-yl)(methyl)diphenylsilane (2) Atomic displacement parameters (Å^2^)

**Table:**

	*U*^11^	*U*^22^	*U*^33^	*U*^12^	*U*^13^	*U*^23^
Si1	0.01095 (11)	0.01317 (12)	0.01416 (12)	0.00328 (9)	0.00118 (8)	0.00181 (9)
O1	0.0202 (3)	0.0199 (3)	0.0313 (4)	0.0113 (3)	0.0083 (3)	0.0103 (3)
C1	0.0112 (3)	0.0134 (4)	0.0177 (4)	0.0040 (3)	0.0002 (3)	0.0020 (3)
C2	0.0156 (4)	0.0227 (5)	0.0198 (4)	0.0084 (3)	0.0040 (3)	0.0054 (4)
C3	0.0176 (4)	0.0309 (6)	0.0272 (5)	0.0104 (4)	0.0078 (4)	0.0051 (4)
C4	0.0194 (4)	0.0225 (5)	0.0318 (6)	0.0116 (4)	0.0051 (4)	0.0019 (4)
C5	0.0114 (3)	0.0150 (4)	0.0170 (4)	0.0042 (3)	0.0020 (3)	0.0036 (3)
C6	0.0144 (4)	0.0176 (4)	0.0169 (4)	0.0044 (3)	0.0023 (3)	0.0015 (3)
C7	0.0150 (4)	0.0186 (4)	0.0217 (4)	0.0044 (3)	0.0004 (3)	−0.0014 (3)
C8	0.0158 (4)	0.0165 (4)	0.0307 (5)	0.0030 (3)	0.0023 (4)	0.0036 (4)
C9	0.0198 (4)	0.0210 (5)	0.0270 (5)	0.0019 (4)	0.0052 (4)	0.0088 (4)
C10	0.0171 (4)	0.0197 (4)	0.0192 (4)	0.0026 (3)	0.0025 (3)	0.0056 (4)
C11	0.0138 (4)	0.0149 (4)	0.0178 (4)	0.0055 (3)	0.0038 (3)	0.0024 (3)
C12	0.0176 (4)	0.0185 (4)	0.0214 (4)	0.0071 (3)	0.0017 (3)	0.0046 (3)
C13	0.0252 (5)	0.0249 (5)	0.0249 (5)	0.0127 (4)	0.0020 (4)	0.0084 (4)
C14	0.0303 (5)	0.0206 (5)	0.0265 (5)	0.0133 (4)	0.0083 (4)	0.0100 (4)
C15	0.0243 (5)	0.0159 (4)	0.0292 (5)	0.0058 (4)	0.0070 (4)	0.0055 (4)
C16	0.0169 (4)	0.0169 (4)	0.0265 (5)	0.0043 (3)	0.0019 (4)	0.0042 (4)
C17	0.0205 (4)	0.0237 (5)	0.0161 (4)	0.0040 (4)	0.0001 (3)	0.0025 (4)

### (4,5-Dihydrofuran-2-yl)(methyl)diphenylsilane (2) Geometric parameters (Å, º)

**Table:**

Si1—C1	1.8743 (10)	C8—H8	0.998 (16)
Si1—C5	1.8720 (9)	C8—C9	1.3854 (17)
Si1—C11	1.8715 (10)	C9—H9	1.023 (16)
Si1—C17	1.8591 (11)	C9—C10	1.3944 (15)
O1—C1	1.3897 (12)	C10—H10	0.960 (15)
O1—C4	1.4603 (13)	C11—C12	1.3988 (14)
C1—C2	1.3356 (14)	C11—C16	1.4050 (14)
C2—C3	1.5082 (15)	C12—H12	0.944 (15)
C2—H2	0.961 (16)	C12—C13	1.3962 (15)
C3—H3A	0.981 (17)	C13—H13	0.928 (16)
C3—H3B	0.933 (18)	C13—C14	1.3861 (17)
C3—C4	1.5398 (18)	C14—H14	0.953 (16)
C4—H4A	0.982 (17)	C14—C15	1.3909 (17)
C4—H4B	0.987 (17)	C15—C16	1.3911 (15)
C5—C6	1.4023 (14)	C15—H15	0.969 (16)
C5—C10	1.4014 (13)	C16—H16	0.990 (16)
C6—H6	0.934 (15)	C17—H17A	0.950 (18)
C6—C7	1.3926 (14)	C17—H17B	0.975 (19)
C7—H7	0.956 (16)	C17—H17C	0.992 (19)
C7—C8	1.3919 (16)		
			
C5—Si1—C1	108.44 (4)	C7—C8—H8	120.4 (9)
C11—Si1—C1	105.50 (4)	C9—C8—C7	119.76 (10)
C11—Si1—C5	113.08 (4)	C9—C8—H8	119.8 (9)
C17—Si1—C1	109.26 (5)	C8—C9—H9	120.5 (9)
C17—Si1—C5	110.48 (5)	C8—C9—C10	120.10 (10)
C17—Si1—C11	109.89 (5)	C10—C9—H9	119.4 (9)
C1—O1—C4	107.48 (8)	C5—C10—H10	121.0 (9)
O1—C1—Si1	117.13 (7)	C9—C10—C5	121.24 (10)
C2—C1—Si1	129.71 (8)	C9—C10—H10	117.7 (9)
C2—C1—O1	113.10 (9)	C12—C11—Si1	123.40 (7)
C1—C2—C3	110.25 (9)	C12—C11—C16	117.71 (9)
C1—C2—H2	123.6 (9)	C16—C11—Si1	118.89 (8)
C3—C2—H2	126.0 (9)	C11—C12—H12	121.5 (9)
C2—C3—H3A	111.2 (10)	C13—C12—C11	121.09 (10)
C2—C3—H3B	112.3 (11)	C13—C12—H12	117.4 (9)
C2—C3—C4	101.51 (9)	C12—C13—H13	118.8 (10)
H3A—C3—H3B	105.5 (14)	C14—C13—C12	120.16 (10)
C4—C3—H3A	112.9 (10)	C14—C13—H13	121.0 (10)
C4—C3—H3B	113.7 (11)	C13—C14—H14	120.8 (10)
O1—C4—C3	106.70 (8)	C13—C14—C15	119.80 (10)
O1—C4—H4A	108.8 (10)	C15—C14—H14	119.4 (10)
O1—C4—H4B	107.0 (10)	C14—C15—C16	119.92 (10)
C3—C4—H4A	111.5 (10)	C14—C15—H15	121.1 (9)
C3—C4—H4B	114.2 (10)	C16—C15—H15	119.0 (9)
H4A—C4—H4B	108.4 (14)	C11—C16—H16	119.7 (9)
C6—C5—Si1	121.29 (7)	C15—C16—C11	121.32 (10)
C10—C5—Si1	120.94 (7)	C15—C16—H16	118.9 (9)
C10—C5—C6	117.64 (9)	Si1—C17—H17A	109.9 (11)
C5—C6—H6	120.3 (9)	Si1—C17—H17B	108.7 (11)
C7—C6—C5	121.26 (9)	Si1—C17—H17C	110.5 (11)
C7—C6—H6	118.4 (9)	H17A—C17—H17B	112.2 (15)
C6—C7—H7	118.2 (10)	H17A—C17—H17C	109.4 (15)
C8—C7—C6	119.98 (10)	H17B—C17—H17C	106.1 (15)
C8—C7—H7	121.8 (10)		
			
Si1—C1—C2—C3	175.12 (8)	C6—C7—C8—C9	1.46 (16)
Si1—C5—C6—C7	174.69 (8)	C7—C8—C9—C10	−1.42 (17)
Si1—C5—C10—C9	−174.66 (9)	C8—C9—C10—C5	0.07 (17)
Si1—C11—C12—C13	178.63 (8)	C10—C5—C6—C7	−1.15 (15)
Si1—C11—C16—C15	−179.42 (8)	C11—Si1—C1—O1	167.77 (7)
O1—C1—C2—C3	−2.00 (12)	C11—Si1—C1—C2	−9.26 (11)
C1—Si1—C5—C6	−51.23 (9)	C11—Si1—C5—C6	65.41 (9)
C1—Si1—C5—C10	124.48 (8)	C11—Si1—C5—C10	−118.88 (9)
C1—Si1—C11—C12	109.11 (9)	C11—C12—C13—C14	0.82 (17)
C1—Si1—C11—C16	−71.17 (9)	C12—C11—C16—C15	0.31 (15)
C1—O1—C4—C3	8.95 (11)	C12—C13—C14—C15	0.27 (17)
C1—C2—C3—C4	7.17 (12)	C13—C14—C15—C16	−1.03 (17)
C2—C3—C4—O1	−9.52 (11)	C14—C15—C16—C11	0.74 (17)
C4—O1—C1—Si1	177.92 (7)	C16—C11—C12—C13	−1.09 (15)
C4—O1—C1—C2	−4.57 (12)	C17—Si1—C1—O1	49.68 (8)
C5—Si1—C1—O1	−70.80 (8)	C17—Si1—C1—C2	−127.35 (10)
C5—Si1—C1—C2	112.17 (10)	C17—Si1—C5—C6	−170.95 (8)
C5—Si1—C11—C12	−9.26 (10)	C17—Si1—C5—C10	4.76 (10)
C5—Si1—C11—C16	170.46 (8)	C17—Si1—C11—C12	−133.23 (9)
C5—C6—C7—C8	−0.15 (16)	C17—Si1—C11—C16	46.49 (9)
C6—C5—C10—C9	1.20 (15)		

### (4,5-Dihydrofuran-2-yl)(methyl)diphenylsilane (2) Hydrogen-bond geometry (Å, º)

**Table:**

*D*—H···*A*	*D*—H	H···*A*	*D*···*A*	*D*—H···*A*
C15—H15···O1^i^	0.969 (16)	2.640 (16)	3.3422 (13)	129.6 (12)
C17—H17*C*···O1^ii^	0.992 (19)	2.584 (19)	3.5168 (14)	156.5 (15)

Symmetry codes: (i) *x*, *y*+1, *z*; (ii) −*x*+2, −*y*+1, −*z*+1.

### Selected geometric parameters for compound 1 (Å ,°)

**Table:**

Si1—C1	1.8742 (3)	C1—Si1—C5	108.189 (12)	
Si1—C5	1.8693 (3)	C1—Si1—C9	106.801 (14)	
Si1—C9	1.8631 (3)	C1—Si1—C10	109.191 (13)	
Si1—C10	1.8579 (3)	C5—Si1—C9	110.770 (15)	
		C5—Si1—C10	108.128 (13)	
C1—C2	1.3370 (4)	C9—Si1—C10	113.628 (16)	
C3—C4	1.5331 (5)			
C5—C6	1.3409 (4)			
C7—C8	1.5298 (5)			
